# Persistent Arteria Hyaloidea or Filaria in the Vitreous Humour of the Eye of the Horse

**Published:** 1884-01

**Authors:** 


### PERSISTENT ARTERIA HYALOIDEA OR FILARIA IN THE VITREOUS HUMOR OF THE EYE OF THE HORSE.

In the left eye of a horse fourteen years old that presented, on the most exact examination nothing pathological, was to be seen a black thread-like object interrupting the continuity of the clearness of the vitreous humor. The cornea of the other eye was completely clouded.

*Leukoma*·—The anterior end of this object was attached to the lens, somewhat below and external to the posterior pole. The thicker posterior end appeared to cease abruptly in a direction towards the optic disk. The object followed the movements of the eye with a sort of worm-like motion. The abscence of every form of clouding, or other pathological changes in the vitreous humor, made it very improbable that this object could have had an inflammatory origin. The circumstance that it was attached to the posterior part of the lenticular capsule made it far more probable that I had to do with a persisting "arteria hyaloidea" than with a dead filaria of any kind. On the other hand we frequently find an arteria hyaloidea in young animals, and I have no doubt that exact opthalmoscopic examination will reveal that the persistence of this object is by no means unfrequent. I will further remark that I have been *very frequently* able to demonstrate the presence of them in young cats during during the first week of life and proved arteria hyaloidea my opthalmoscopic diagnosis by an anatomical examination, so that I consider the same a normal attribute of the eye up to the second week or so of life.

[These three accounts of opthalmoscopic appearances in the eye are of great practical and diagnostic value to the practitioner, and occasions might arise where they would not be without value from a forensic point of view. It is important to know that the sight is not interfered with by the presence of double contoured fibres in the papilla of the optic nerve. They are congenital formations as well as the cholesterin crystals in the vitreous humor and the arteria hyaloidea persistens. The last two phenomena cause no disturbances in the sight of the human eye worthy of mention, and we have also a right to consider them of the same nature in animals.—Ed.]

